# Special Section Guest Editorial: Thirty Years of Functional Near-Infrared Spectroscopy

**DOI:** 10.1117/1.NPh.10.2.023501

**Published:** 2023-07-06

**Authors:** David Highton, David Boas, Yasuyo Minagawa, Rickson C. Mesquita, Judit Gervain

**Affiliations:** a Princess Alexandra Hospital Southside Clinical Unit, University of Queensland, Woolloongabba, Queensland, Australia; b Boston University Neurophotonics Center, Boston, Massachusetts, United States; c Keio University, Department of Psychology, Yokohama, Japan; d University of Campinas, Institute of Physics, Campinas, Brazil; e Université Paris Cité, Integrative Neuroscience and Cognition Center, CNRS, Paris, France; f University of Padua, Department of Developmental and Social Psychology, Padua, Italy

## Abstract

Functional Near-Infrared Spectroscopy (fNIRS) is a non-invasive optical technique that measures cerebral hemodynamics across multiple regions of interest, and thereby characterises brain functional activation. Since its first description in 1993, fNIRS has undergone substantial developments in hardware, analysis techniques, and applications. Thirty years later, this technique is significantly enchancing our understanding in diverse areas of neuroscience research such as neurodevelopment, cognitive neuroscience, psychiatric disorders, neurodegenerative conditions, and brain injury management in intensive care settings. This special issue outlines the latest progress in instrumentation and analysis techniques and showcases some applications within the expanding field of fNIRS over the past decade.

## 1 Introduction

Functional near-infrared spectroscopy (fNIRS) is an optical technique that enables non-invasive monitoring and imaging of cerebral hemodynamics, oxygenation, and metabolism to evaluate brain function in health and disease states.^1^ Near-infrared light (∼700−900  nm wavelength) penetrates several centimetres through biological tissue, due to the relative transparency of tissues in this region of the electromagnetic spectrum. Light emitted by a source optode placed on the head diffuses through cerebral structures, including the surface of the cerebral cortex, where it undergoes scattering and absorption interactions with the tissue. Part of the scattered light can be measured by one or more detectors several centimeters away from the source and used to assess tissue properties, most notably the concentration of hemoglobin species (oxy/deoxy-hemoglobin), by measuring the attenuation of light across multiple wavelengths. By combining arrays of sources and detectors, it is now feasible to reconstruct not only topographic, but also tomographic images of these properties.^2^ Functional activation within specific brain areas induces a functional hyperemia, resulting in a characteristic hemodynamic response function with increased flow, oxy-hemoglobin levels, and reduced deoxy-hemoglobin concentrations.^3^

fNIRS hardware offers distinct advantages for functional neuroimaging over other techniques. fNIRS is non-invasive, non-ionizing, inexpensive, portable, and can be used in mobile individuals and dynamic environments in real time with high temporal resolution. This lends itself to situations where other conventional imaging techniques are challenging to use. With the microelectronics revolution observed in the past decade, fNIRS has found increasing use as a functional neuroimaging technique, with particular advantages in studying brain activity unrestricted in a natural interactive environment. In addition, pediatric studies benefit from comfort and unrestricted movement offered from current fNIRS devices. Environment compatibility means that fNIRS can be performed in intensive care or the operating room, or alongside other modalities such as electroencephalography without interference or safety issues.

Since the first descriptions of fNIRS in 1993,^4^ hardware has substantially advanced, accompanied by a broad expansion of research applications and emerging clinical utilization. Fast forward 20 years to 2013 and fNIRS had blossomed into a mature experimental technique. These achievements were celebrated in a special issue describing the state of the art in fNIRS.^5^ In the past 10 years, the field has experienced continued innovation and consolidation of hardware, analysis techniques, and applications. Publication continues to grow, with approximately 300 papers per year being published.^6^ Key developments in hardware miniaturization, analysis standardization, hardware accessibility and transition to imaging have enabled fNIRS to consolidate its position as a reliable and practical research tool. These advancements lay the foundation for a bright future in clinical and neuroscience applications.

This special issue celebrates 30 years of fNIRS summarizing key progress in: instrumentation (wearable high density optical imaging, speckle contrast, and interferometric techniques), fNIRS methods and modelling, and specific fNIRS applications (neuro development, clinical applications, neuro degeneration and psychiatric applications). This is formed by review articles and exemplar research, grouped into various topics as collected here and as summarized below.

## 2 Instrumentation

Advances in instrumentation aim to provide researchers and clinicians with devices that offer enhanced spatiotemporal resolution, depth sensitivity, comfort, ease of use, portability, affordability, and deliver clinically relevant measurements.

The last 10 years have seen remarkable advances in optoelectronics, which have facilitated development of wearable high density optical imaging. In this issue, Vidal-Rosas and colleagues^2^ describe recent advances in wearable high-density optical tomography. Devices now have the capacity to achieve resolution comparable to functional magnetic resonance imaging, yet remain wearable and allow normal movement and interaction with the environment. When paired with image reconstruction techniques, these can generate tomographic images that parallel those of magnetic resonance imaging. However, this creates an ill posed mathematical problem and advances in analysis have been essential to improve performance.^7^

A range of optical techniques have been used for fNIRS, including continuous wave, frequency domain, time domain, and broadband spectroscopy. Recent advances also allow measuring cerebral blood flow using diffuse correlation spectroscopy and interferometric techniques.^8^^,^^9^ Interferometric techniques offer some hardware advantages over diffuse correlation spectroscopy, and both have progressed to multichannel systems that can reconstruct brain blood flow maps. Direct measurement of blood flow has theoretical advantages over measuring surrogates such as concentration of hemoglobin species. Combined with measures of oxygenation this offers measurements of metabolism by calculating the cerebral metabolic rate of oxygen.

## 3 fNIRS Methods and Modeling

Improvement in experimental design, analysis techniques, and reporting has been a key focus for progress in fNIRS research. Standards for reporting, preregistration,^10^ and open science practices with data sharing continue to increase.^11^ Data sharing is central to validating research findings and performing secondary analyses. Open source analysis packages and data formats (e.g., SNIRF) are facilitating this practice. fNIRS analysis is particularly sensitive to confounding from systemic physiology and contamination of signal from superficial extracranial tissues. Sophisticated analysis techniques continue to evolve to tackle these issues.^12^^–^^15^

Defining key experimental events for analysis, whether physiological for removal or events such as tasks or social interaction, is critical to interpret data. Xu et al. describe automated social interaction detection to characterize fNIRS responses in a natural environment.^16^

## 4 fNIRS Applications

fNIRS is increasingly used for neurodevelopmental studies due to its many favorable characteristics for use with infants and children. Gervain et al. review recent advances and future directions.^17^ Current trends include an increasing global health presence, performing investigations in a natural environment, hyper scanning and application of new hardware and analysis techniques, such as meta-analyses.^18^ This is revealing insight into both the normal development of brain function and atypical development such as autism and attention deficit disorder.

Clinical applications for fNIRS show considerable promise and it is on the verge of being a useful clinical tool in a range of settings. Gallagher et al. review pediatric applications.^19^ fNIRS has many advantages in this setting due to comfort, tolerance of movement, and ability to use in a natural environment. Particular applications include epilepsy, communicative disorders, and attention deficit disorder.

In the adult population, fNIRS has advantages in the intensive care and operating room monitoring at the bedside where other imaging modalities such as magnetic resonance imaging are incompatible. Thomas et al. review the current state of fNIRS in intensive care.^20^ In brain injury requiring intensive care there is a strong physiological rationale for monitoring cerebral hemodynamics and metabolism to identify and treat ischemia. Instrumentation such as combined diffuse correlation spectroscopy and fNIRS is particularly appealing as it can characterize flow, oxygenation, and thereby metabolic rate. Monitoring cerebral autoregulation of blood flow holds considerable potential as a clinical treatment target to optimize blood pressure.

Adult applications also include neurodegeneration and psychiatric applications. Srinivasan et al. review advances in fNIRS for investigating dementia which have vascular and metabolic dysfunction as key pathophysiological features.^21^ fNIRS is useful in this group because of tolerance to movement and use in the natural setting for function tasks to characterize disease. Li et al. review fNIRS progress in psychiatry applications where it is used to study functional activation in diverse disorders including schizophrenia, depression, bipolar disorder, and obsessive-compulsive disorder.^22^ Exciting prospects exist to use fNIRS as a neuro-feedback tool as a therapy. NIRS may also be applied to measure the effects of psychedelic substances.^23^

Further, NIRS also offers to be a promising tool to assess the neural correlates of how medical professionals gain expertise with new surgical skills and techniques, making NIRS a useful method not only for patient assessment, but also for medical training.^24^

In conclusion, we believe that this special issue represents a milestone in the development of fNIRS at its mature age of 30. The early years focused on establishing its safety and feasibility, followed by successful translation to neuroimaging applications. Now, we have reached a stage where fNIRS can thrive in unforeseen and diverse scenarios that were unimaginable just a decade ago. This has led to exciting discoveries in various research and clinical domains. As we embark upon a new decade, we eagerly envision the promising and transformative years that lie ahead.
